# Contextual memory reactivation modulates Ca^2+^-activity network state in a mushroom body-like center of the crab *N. granulata*

**DOI:** 10.1038/s41598-022-15502-1

**Published:** 2022-07-06

**Authors:** Francisco Javier Maza, Francisco José Urbano, Alejandro Delorenzi

**Affiliations:** 1 Instituto de Fisiología, Biología Molecular y Neurociencias (IFIBYNE-UBA-CONICET), Ciudad Universitaria, Pabellón IFIBYNE, Buenos Aires, Argentina; 2 Departamento de Fisiología, Biología Molecular y Celular, Facultad de Ciencias Exactas y Naturales, Universidad de Buenos Aires, Ciudad Universitaria, Buenos Aires, Argentina

**Keywords:** Learning and memory, Fluorescence imaging

## Abstract

High-order brain centers play key roles in sensory integration and cognition. In arthropods, much is known about the insect high-order centers that support associative memory processes, the mushroom bodies. The hypothesis that crustaceans possess structures equivalent to the mushroom bodies -traditionally called hemiellipsoid body- has been receiving neuroanatomical endorsement. The recent functional support is limited to the short term: in a structure of the true crab *Neohelice granulata* that has many insect-like mushroom bodies traits, the plastic learning changes express the context attribute of an associative memory trace. Here, we used in vivo calcium imaging to test whether neuronal activity in this structure is associated with memory reactivation in the long-term (i.e., 24 h after training). Long-term training effects were tested by presenting the training-context alone, a reminder known to trigger memory reconsolidation. We found similar spontaneous activity between trained and naïve animals. However, after training-context presentation, trained animals showed increased calcium events rate, suggesting that memory reactivation induced a change in the underlying physiological state of this center. Reflecting the change in the escape response observed in the paradigm, animals trained with a visual danger stimulus showed significantly lower calcium-evoked transients in the insect-like mushroom body. Protein synthesis inhibitor cycloheximide administered during consolidation prevented calcium mediated changes. Moreover, we found the presence of distinct calcium activity spatial patterns. Results suggest that intrinsic neurons of this crustacean mushroom body-like center are involved in contextual associative long-term memory processes.

## Introduction

The insect mushroom bodies (MBs) are higher order brain centers involved in multi-sensory integration and associative memory processes^1^. Hemiellipsoid bodies has been used as the traditional denomination for the proposed high-order integration center in crustaceans’ central nervous systems, processing inputs from different sensory modalities. Previous studies have suggested that the crustaceans' higher order centers might be structures with equivalent neuroarchitecture and cognitive functions to insect MBs. Essentially, insect MBs are composed of small association intrinsic neurons (globuli or Kenyon cells) that typically form calyces with their dendrites and project parallel axon-like processes (the pedunculus) forming a system of lobes, the major output regions^1–4^. Vast number of studies supports, as Kenyon has suggested more than a century ago, that insects MBs play central roles in associative memories processes of different complexity^1,5–8^. A key feature of the MBs is their attribute to act as a re-coding center, converting sensory information to value-based information. Thus, it was pointed out that MBs shares functional properties with the mammalian hippocampus and prefrontal cortex^1^. The developmental and neuroarchitectural similarities between the pallium of vertebrates and the MBs of annelids and insects, support deep homology that dates the origin of higher brain centers back to the time of the protostome-deuterostome ancestor^9^.

In a recent article^10^, the long-standing discussion regarding possible genealogical relationships between insect MBs and malacostracan crustacean hemiellipsoid bodies was renovated based on newly obtained data^2,11–13^. According to previous suggestions^10,11^, we hereafter use the term MBs for these high-order centers of the protocerebrum in crustaceans. Crustaceans MBs appeared to share a common ground-plan that has been described in insects and in other invertebrate phyla^4^ (e.g., Annelida^14^), displaying a variety of changed morphologies and sizes^10,12^. Although crustaceans’ MBs were highly modified along evolution and present variable morphologies^10,12^, a set of morphological evidence supports that are equivalent to those of insects^10,12^. Several reports support the centennial proposition that MBs of crustacean would be high-order integration centers with indirect sensory input and involved in multimodal integration, based on the identification of neuronal activity from both olfactory lobes and visual integration neuropils^12,15–18^. Crustacean’s MBs hypothesized roles on associative learning and memory have been inferred from comparative neuroanatomical studies, including the identification of expression of proteins involved in long-term memory formation and adult neurogenesis^10,13,18–20^.

The first evidence in favor of the occurrence of associative learning and memory processes in a crustacean’s MBs was found on the crab *Neohelice granulata* (Dana, 1851) (Brachyura: Grapsoidea: Varunidae)^17^. As an experimental model, crab *N. granulata* have been extensively used to accumulate research on the neurobiology of memory^21,22^. Recent functional and morphological evidence support that the *N. granulata* high-order brain center (hemiellipsoid body)^19,20,23–26^ can be interpreted as an equivalent structure to the insect MBs, i.e., a *mushroom body-like structure (MB-ls)*^17^. A counterview was introduced by other authors^27–29^, who have recently proposed that true crabs possess novel, inverted, and highly modified MBs with an expansive system of gyri^27^. The above-mentioned neurophysiological evidence^17^, showing that the associative context attribute of an aversive memory trace was present in the crabs’ MB-ls, is restricted to the short-term. However, in this work our data further support our previous prediction suggesting long-term memory reactivation can be triggered by the presentation of the training context in the *N. granulata’s* MBs-ls.

Extensive research by other groups have shown spontaneous brain activity (i.e., an activity that is not evoked by the explicit presentation of stimuli)^30^ as a ubiquitous phenomenon. By continued adjusting endogenous brain states, spontaneous brain activity would orchestrate neuronal activity^31^ and could play a role in a myriad of cognitive process, including the retrieval of spatial memories^32^. Like vertebrate pallium structures, insects’ MBs circuits presented spontaneous activity that was modulated during cognitive processes^30,33–35^. In insects, the MBs responses to contextual inputs enable flexible responses based on past experiences^1^. Changes in spontaneous brain activity have been envisioned to play a role by tuning endogenous brain states during the processing of exogenous information^30,33–35^. In the *N. granulata* crab aversive paradigm, memory results from an association between the training context and a visual danger stimulus (VDS) that resembles an aerial predator^21^. During strong (15-trials) training, crab’s escape response decreases, and a freezing response progressively replaced it. This memory is dependent on the context in which the animal was tested and last for up to four days after training. Studies at behavioral, anatomical, and cellular levels have provided an integrated description of different memory phases of a contextual-associative memory in Neohelice granulata. Numerous characteristics of acquisition, consolidation, extinction, retrieval and memory expression, and reconsolidation phases have been described^21,36–56^. Such experimental paradigm has contributed to describe one of the conditions that initiates the updating of consolidated memories during a process called reconsolidation. Consolidated memories (in the presence or absence of behavioral expression) were described to enter a *labile-state* after a reminder that contains a mismatch^50,56–61^. Here, we used in vivo calcium imaging in a MB-ls of *N. granulata* to assess whether spontaneous activity is modified by the presentation of a reminder structure that triggers the reconsolidation process^22^ of an aversive long term (i.e., 24 h after) memory.

Our results suggest that in the MB-ls of the crab *N. granulata* the presentation of a reminder, known to initiate the reconsolidation processes of the reactivated memory, induced an increase in spontaneous Ca^2+^ activity. Changes in Ca^2+^ activity were triggered by a trained visual danger stimulus, which furthermore correlated to the changes in spontaneous Ca^2+^ activity, ultimately suggesting reactivation of the associative long-term memory trace in the MB-ls.

## Materials and methods

### Animals

Intermolt adult male crabs of the species *Neohelice granulata* measuring between 2.7 and 3.0 cm across the carapace (average weight 17 g) were collected from the narrow coastal inlets of San Clemente del Tuyú, Buenos Aires Province, Argentina. In the laboratory, crabs were kept on a 12:12 h light–dark cycle, in collective plastic tanks (20 animals each) filled up to 2 cm depth with brackish water prepared with Coral Pro Salt (Red Sea, Israel) 1% (m/m), pH 7.4–7.6. The holding and experimental rooms were kept at 22–24 °C and 80 ± 10% relative humidity. Procedures were carried out at daytime, within fifteen days after the animals had arrived at the laboratory. All efforts were made to minimize the number of animals. The research was conducted in accordance with the Ethical Reference Frame for Biomedical Investigations of CONICET, equivalent to the standard procedures for animal care and use of the NIH of the U.S.A.

### Anesthesia

Before invasive procedures, crabs were cryo-anesthetized by submerging them for 3–5 min in brackish water in equilibrium with ice^62^. Anesthetized animals were held partially submerged in the same ice-cold brackish water during the surgery and staining.

### In vivo calcium imaging preparation

**Figure 1:**
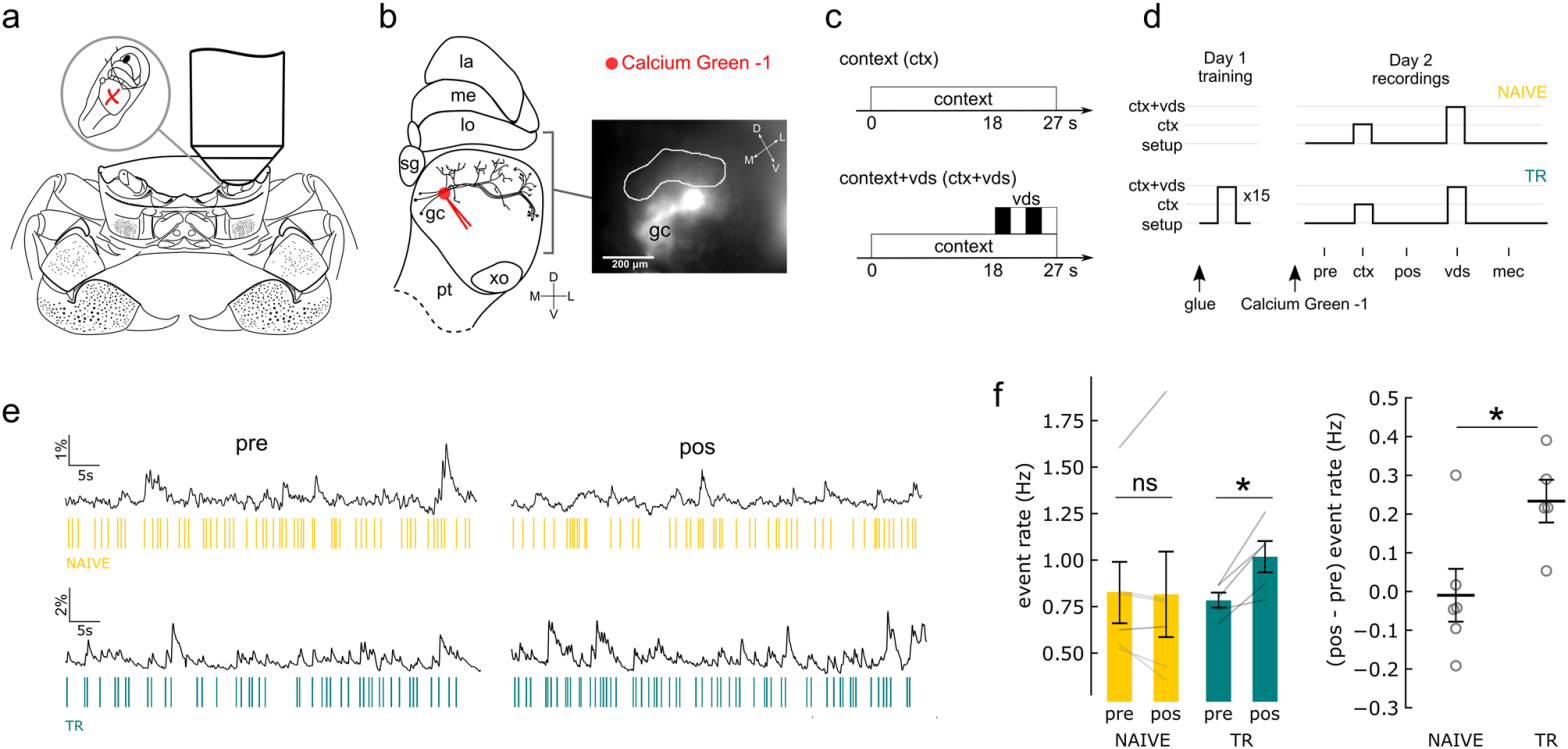
Presentation of the training context induced an increase in the Ca^2+^ event rate in crabs trained 24 h before. (**a**), *Neohelice granulata* crab and detail of the left eyestalk. The mushroom body-like structure (MB-ls) is in the lateral protocerebrum (marked with a red cross). Fluorescence recordings were done in the left eyestalk. (**b**), Left, frontal view of neuropils in the eyestalk. For in vivo Ca^2+^ imaging, a window was opened in the left eyestalk and a calcium sensitive dye (Calcium Green-1 dextran) was inserted in the tract of the MB-ls globuli cells. Right, example of the preparation view under the microscope. The bright spot corresponds to the place where the dye crystals were stabbed. Orientation references match an upright eyestalk position. The region of interest (ROI) is delimited. (**c**), Context and vds trials scheme. Context presentation involves a phasic change in setup illumination. The visual danger stimulus (vds) is presented during the last nine seconds of a 27 s context presentation and comprises a black rectangular panel moving over the horizon of the crab back and forth twice in each trial (vds, black bars). (**d**), Experimental protocol. On day one, crabs have their left eyestalk glued in place ready to be open for recording at day two. Then, they were trained (TR group) under the microscope with of the vds over the crab's right eyestalk (15 trials, ISI 3 min). Naive group (NAIVE) was not trained. Each visual stimulus was preceded by the onset of the context (see “c”). On day two, Calcium Green -1 dextran was inserted and several recordings at different periods were done. Recordings involved periods of no stimulation (pre, pos) and trials with context, visual or mechanical stimulation (ctx, vds and mec). (**e**), Examples of ΔF/F (%) obtained during pre and pos context presentation periods for a NAIVE (above) and TR (below) animal. Ticks below each curve correspond to Ca^2+^ events. (**f**), Event rate (Hz) for NAIVE and TR animals during pre and pos periods (left) and difference between pos and pre period (right). Grey lines and circles correspond to individual animals. Means ± sem are shown. Post hoc Welch two-tailed paired t-tests and independent t-test, *p < 0.05, *ns* not significant. Abbreviations: *gs* sinus gland; *gc* globuli cells; *pt* protocerebral tract; *lp* lateral protocerebrum; *xo* X-organ; *D* dorsal; *L* lateral; *V* ventral; *M* medial.

The in vivo optical imaging method was based on our group’s previously described^17,45^. Recordings were made from the left eyestalk's mushroom body-like structure (hemiellipsoid body) bulk stained with a calcium sensitive dye, while the right eye remained open to visual stimulation (Fig. 1a). On day 1, the crabs were anesthetized, the chelae were immobilized with a rubber band and the crabs were firmly held by their exoskeleton with an adjustable clamp. The right eyestalk was covered with wet paper to prevent visual stimulation during the preparation. This cover was removed once the animal was set under the microscope ready for the experiment. Left eyestalk was fixed with acrylic glue (*La Gotita*, Akapol S.A., Argentina) oriented to allowed access to the anteromedial side of the eyestalk. A small container was built around eyestalk with dental cement (Dycal, Dentsply International Inc.). This container allows a continuous flow of the crab Ringer's solution (468.00 mM NaCl, 9.46 mM KCl, 7.50 mM MgCl_2_, 12.53 mM CaCl_2_, 5.00 mM Hepes, and 2 mM glucose, pH 7.75) that is necessary once the cuticle is removed at day 2. Animals were allowed to recover from the anesthesia for 40–60 min prior to their setting in the microscope for training. One day 2, crabs were also anesthetized and immobilized. Right eyestalk was covered again with wet paper to prevent visual stimulation during the preparation. A window (circa 3 × 2 mm) was opened in the cuticle of the already fixed left eyestalk using a sharp scalpel. The opened surface was covered with crab Ringer’s solution. To allow visual access to the neuropiles, the connective tissue was removed and neuroepithelium cut along the longest axis of eyestalk with vannas scissors (WPI 555640S) and attached to the edges of the window. Calcium Green-1 dextran crystals (Potassium Salt 3000 MW Anionic; Molecular Probes, Life Technologies, Cat#C6765) were placed into the target tissue by hand using borosilicate glass electrodes (outer diameter = 1.2 mm, inner diameter = 0.69 mm, length = 100 mm; BF120-69-10, Sutter Instruments) that were pulled to obtain a thin tip where a small crystal of the calcium-sensitive dye was attached. Dye crystals were then allowed to dissolve for a few seconds in the MB-ls globuli cells tract, near the somata cluster (Fig. 1b). Excessive dye was then washed using crab Ringer’s solution. Imaging fluorescence recordings started circa 1.5 h afterwards. This preparation requires a physical connection between our microscope objective and the opening performed near the crab’s eye, thus the animal ability to move is greatly limit and leg movements in an attempt to escape the VDS seldom happen in its holding position (i.e., above ground)^63^. However, it is worth mentioning that crabs receiving a training protocol while being physically restrained can learn and exhibit normal long-term memory^64^.

### Data acquisition and processing

Acquisition and stimuli were controlled using Micro-Manager software^65^ and an Arduino board controlled with the Micro-Manager Arduino adapter. Recordings were carried out using a Nikon E600 microscope; a 10X, 0.3 N.A. water immersion objective (Nikon) and a Hamamatsu ORCA-Flash 4.0v2, 16-bit camera. Light source came from a cyan LED (peak ~ 505 nm, spectral width measured at half peak 30 nm; TOLKET S.R.L, Argentina), excitation filter 440-490 nm, dichroic mirror 525 nm, and emission filter 530 nm long-pass. Video recordings were obtained at a frame rate of 20 Hz. Sensor pixels were binned on-chip in 4 × 4 mode, resulting in a recorded pixel size of ~ 2.3 × 2.3 μm. Exposure time was equal to the frame interval (50 ms) and excitation LED intensity was set to obtain fluorescence values in the dynamic range of the acquisition camera. Continuous recordings lasted up to a maximum of 400 s. Motion artifacts in the x–y axes were reduced using the Fiji/ImageJ Image Stabilizer plugin^66^. When motion artifacts were unfixable, segments or full recordings were discarded.

### Stimuli and contexts

In the training protocol used here, originally denominated contextual Pavlovian conditioning^36,44,45,67^, phasic presentation of the training context was achieved by changing superior background lights in the setup^17,36^. Setup condition consists of illumination underneath the white container where the crab is semi submerged (i.e., below light), and results in a dim illumination of the setup. Plain white walls surround the animal's field of view. Training context is active when the setup illumination changes by turning off the below light and turning on a light that hits context walls. This paradigm is interpreted as a contextual conditioning phenomenon; for instance, crabs recognized both illumination settings as different contexts^36,68^. In addition, the above illumination setting does not play a role as a cue with the VDS (the proposed US)^36,44^. A schematic cartoon of the recording setup is shown in Supplementary Information 1 Fig. S1.

Visual stimulus is based on the visual danger stimulus (VDS) used in the *N. granulata* memory paradigm^21,40^. It involves a black opaque rectangle (7.5 × 2.25 cm) that moves in a 90° clockwise and counterclockwise excursion from the starting position and back (~ 2.2 s). While the training context can be presented solely (“ctx”), the vds trial requires the concomitant ctx to be evaluated (“ctx + vds”), thus each vds trial comprised two movement cycles separated by 2 s at the end of 27 s of active training context (Fig. 1c,d). Mechanical stimulus (MEC) comprised by a pulse of nitrogen gas (10 psi) delivered using a PV830 Pneumatic Picopump (World Precision Instruments) by positioning a tip (1 mm aperture) at 0.5 cm of the mid-right dorsal carapace backside. Each MEC trial comprised pulses ranging 1 to 2 s.

### Training and recording protocols

On day 1 animals have their left eyestalk fixed and prepared as it was previously described^17,45^. Experiments were performed inside a cage covered with black cloth to prevent undesired visual stimulation. During recordings, crabs had their chelae immobilized with a rubber band and they were held with half of their body submerged in a white plastic container with brackish water. Recorded neuropils had continuous superfusion of crab Ringer's solution. Protocols began after the animal had remained visually undisturbed for 15 min in the setup. Trained animals (TR group) were placed in the microscope setup and underwent a training protocol that consisted in 15 VDS trials with an interstimulus interval of 180 s. After training they were released and set in drawers in individual containers with brackish water until day 2. As a control, we chose naïve animals to increase the contrast between treatments. Naïve animals (NAIVE group) received the same treatment except for the training session, as they were placed directly in the individual containers after they recovered from the preparation. On day 2 and after recovery from the calcium sensitive dye preparation, NAIVE and TR animals were placed in the setup and calcium activity from the MB-ls was recorded at different periods (Fig. 1d): “pre”: period before training context presentation (3–12 min before context presentation), “ctx”: presentation of the training context, “pos”: period after “ctx” (0.5–12 min after context presentation), “vds”: visual danger stimulus trial (1.5–18 min after “pos”), “posvds”: period after vds trial (0.5–5 min after “vds”), and “mec”: mechanical stimulation (3–22 min after “posvds”). For analysis, we considered continuous recording segments not affected by motion, thus segments timing between animals varied. Analyzed segments for pre, pos and posvds spontaneous activity has an average length of 139 s (min–max range, 60–188 s). In one untrained animal, mec trial was before “vds”. In another untrained animal, “vds” trial was presented previous to “ctx” (excluding this animal from analysis did not change the conclusions).

### Cycloheximide systemic administration

The protein synthesis inhibitor Cycloheximide (CHX) (Sigma-Aldrich, St. Louis, MO, USA, Cat #C7698) was administered at a final dose of 40 μg/crab (circa 2.35 μg/g). Injections were carried out immediately after the training session (see Supplementary Information 1 Fig. S5); in previous studies in Neohelice, memory was shown to be sensitivity to CHX in both consolidation and reconsolidation^36,44,50,52,69^. Fifty microliters (50 μl) of drug solution dissolved in crab’s Ringer solution were administered through the right side of the dorsal cephalothoraxic-abdominal membrane, using a syringe fitted with a sleeve to control the depth of penetration to 4 mm, thus ensuring that the injected solution was released in the pericardial sac.

### Data analysis

#### Relative changes in fluorescence

A region of interest (ROI) on selected MB-ls calyces was chosen for each animal. This ROI was delimited manually based on previously described known morphology^18^ and on the activity elicited during visual and mechanical stimulation. Because of the stochastic nature of the bulk staining used in this preparation, ROIs shape vary between animals (Supplementary Information 1 Fig. S2). We calculated the fluorescence for each ROI in arbitrary units (16 bit, i.e., 0–65,535 range) using Fiji/ImageJ custom written macros. Fluorescence dynamics were got by calculating the average intensity inside the ROI for each video frame (F). Custom written scripts in Python were subsequently used for analyses. Mean fluorescence profiles were band pass filtered (0.025–5 Hz) to reduce noise levels and drift changes observed on baseline fluorescence (i.e., changes in the fluorescence associated with dye bleaching, etc.). For each analyzed recording segment, relative fluorescence changes (%ΔF/F) were computed as (F−F_0_/F_0_)*100, where the baseline F_0_ was estimated as the 8th percentile value over each entire recording period. We opted to use this F_0_ value because spontaneous activity is present throughout the recording period, and slow drifts were already bandpass filtered.

#### Calcium events

Calcium events candidates in %ΔF/F traces were visually identified in all recording segments available for each animal (“pre”, “ctx”, “pos”, “vds”, “posvds”, and “mec”). Candidates ought to present a quick rise in fluorescence followed by exponential-like decay or a short decay followed by a superimposed new event. From these visually recognized candidates we kept as events those that have at least two frames (100 ms) to their peak and a relative amplitude of at least 0.02% ΔF/F and 0.3 of the %ΔF/F standard deviation of all segments for that animal. Peaks were computed as the first peak to occur during a lapse of 2 s after event onset.

#### Spatial activity patterns

Spatial activity patterns inside the ROI for the spontaneous or evoked events were computed in ImageJ/Fiji. For each analyzed segment, recordings were pixel binned (2 × 2) and normalized as %(F−F_0_)/F_0_ (%ΔF/F), where F corresponds to the pixel value and F_0_ to the pixel rolling mean value of 400 frames (20 s), implemented pixel wise with the “Mean 3D” Fiji filter. The minimum value from the %ΔF/F was subtracted from the video; thus, all pixels conserved their intensity relationship but are all >  = 0. Spatial patterns images correspond to the average of the peak frame ± 1, normalized to the maximum pixel value inside the ROI. Thus, patterns considered pixel relative but not absolute intensities. Peak frames correspond to peaks as described previously in Calcium events; for stimuli events, peaks correspond to the first peak after the stimulus was triggered. To increase clustering performance, patterns were gaussian filtered (sigma = 1) and only values >  = 0.5 were kept, except for one animal where performance was better without gaussian filter. Pattern clustering was performed with the K-Means clustering function from the *scikit-learn* package for python^70^ considering the patterns found in all analyzed segments for each animal.

### Statistics

No statistical methods were used to predetermine sample sizes which are similar to those reported in previous publications^17,45,71^.

For evaluation of Ca^2+^ transients triggered by stimuli, we tested for a significant increase in fluorescence during stimuli presentation compared with basal activity, i.e., activity immediately before the stimulation. For vds, we considered the summation of %ΔF/F during the 9 s of stimuli versus the summation of %ΔF/F during the previous 9 s. For mec, we considered the summation of %ΔF/F during the 1 s of stimulus versus the summation of %ΔF/F during the previous 1 s.

Mixed ANOVA was used as an omnibus test, followed by pairwise t-tests. For multiple t-test comparisons, the probability values were compared with α = 0.05 adjusted with a Holm-Bonferroni correction to deal with familywise error. One-tailed paired *t*-tests were considered for transients triggered by stimuli, as an increase in activity is expected. Otherwise, two-tailed *t*-tests were used. When groups sample sizes were not equal, *t*-tests were Welch’s adjusted.

ANOVA and t-tests were done using the Pingouin Python library^72^. Pearson correlation was done using the Scipy Stats module for Python^73^.

## Results

### Spontaneous Ca^2+^ events increased after long-term contextual memory reactivation

*N. granulata* crabs trained using repeated visual stimuli (visual danger stimulus, vds) passing over their visual horizon is known to induce an associative long-term memory, which can be characterized context specific, change in behavior. Such behavioral response is based on an innate escape response that can turns into a training-induced freezing long-term response^21,39^. A training comprising 15 vds (interstimulus interval: 3 min) induced both short- and long-term memory^21,36^. Within the MB-ls^17^, training induced a context-dependent reduction of Ca^2+^ transients triggered by the trained vds when evaluated in the short term (i.e., 30 min after training). Here, we studied whether MBs activity does also reflect the reactivation of a long-term memory trace when crabs were reinstalled in their training context. We ran in vivo Ca^2+^ imaging in the MB-ls by bulk staining of its globuli cells with a calcium sensitive dye (Fig. 1a,b). Trained (TR) and untrained animals (NAIVE) were compared. For each training trial, the vds was preceded by the discrete presentation of the training context (ctx), comprising a change in the setup illumination (see Materials and methods) (Fig. 1c and Supplementary Information 1 Fig. S1).

We first investigated whether the presentation of the training context, a reminder known to induce memory reconsolidation (i.e., memory enters a transient labile state during which can be positively or negatively interfered)^36,50,58^, induced changes of spontaneous activity for animals trained 24 h earlier. Relative changes in fluorescence (%ΔF/F) were evaluated in a region of interest (ROI) in the crab's mushroom body calyx-like regions (Fig. 1b) delimited for each animal (ROI mean µm^2^ ± sem; 42,195.24 ± 3951.31). Supplementary Information 1 Fig. S2 shows the ROI selected for each animal. Both trained (TR) and untrained (NAIVE) animals displayed spontaneous Ca^2+^ activity when in the recording setup, as observed in “pre” context period registers (Fig. 1e; full segment traces are shown at Supplementary Information 2 Figs. S1–2). After the presentation of the training context (“ctx”), no change was observed for the spontaneous activity (“pos'' context period) in NAIVE animals, but an increase in the event rate was observed in TR animals (Two-way 2 [group: NAIVE, TR] × 2 [period: pre, pos] mixed ANOVA; group: F(1,9) = 0.13, *p* = 0.72; period: F(1,9) = 5.07, *p* = 0.051; group-period interaction F(1,9) = 7.33, *p* = 0.024; post hoc *pos* vs. *pre*: NAIVE t(5) = − 0.14, *p* = 0.89; TR t(4) = 4.20, *p* = 0.027) (Fig. 1f). An independent *t*-test also reported an increase in events rate for the TR group when analyzing *pos* minus *pre* rate (Welch t-test t(8.94) = 2.77, *p* = 0.021) (Fig. 1f). To evaluate whether this increase also entailed modifications in the amplitude of the calcium responses, events amplitudes throughout *pre* and *pos* periods were also analyzed; no differences comparing *pre* and *pos* periods were found (pos minus pre mean %ΔF/F amplitude ± sem; NAIVE: 0.046 ± 0.029; TR: 0.010 ± 0.009; NAIVE vs. TR Welch t-test t(5.89) = − 1.18, *p* = 0.28). During the presentation of the training context (“ctx”), no change was observed for the spontaneous activity (vs. “pre'' context period) in both NAÏVE and TR animals (Two-way 2 [group: NAIVE, TR] × 2 [period: pre, ctx] mixed ANOVA; group: F(1,8) = 0.03, *p* = 0.86; period: F(1,8) = 0.04, *p* = 0.84; group-period interaction F(1,8) = 1.15, *p* = 0.31); independent *t*-test *ctx* minus *pre* rate (Welch t-test t(5.44) = 1.02, *p* = 0.35) (Supplementary Information 1 Fig. S3). These results suggest that, in the MB-ls, a context-reactivation of this aversive long-term memory induced a change in the spontaneous activity rate.

### Ca^2+^-transients induced by visual danger stimuli were reduced for animals trained 24 h earlier

**Figure 2:**
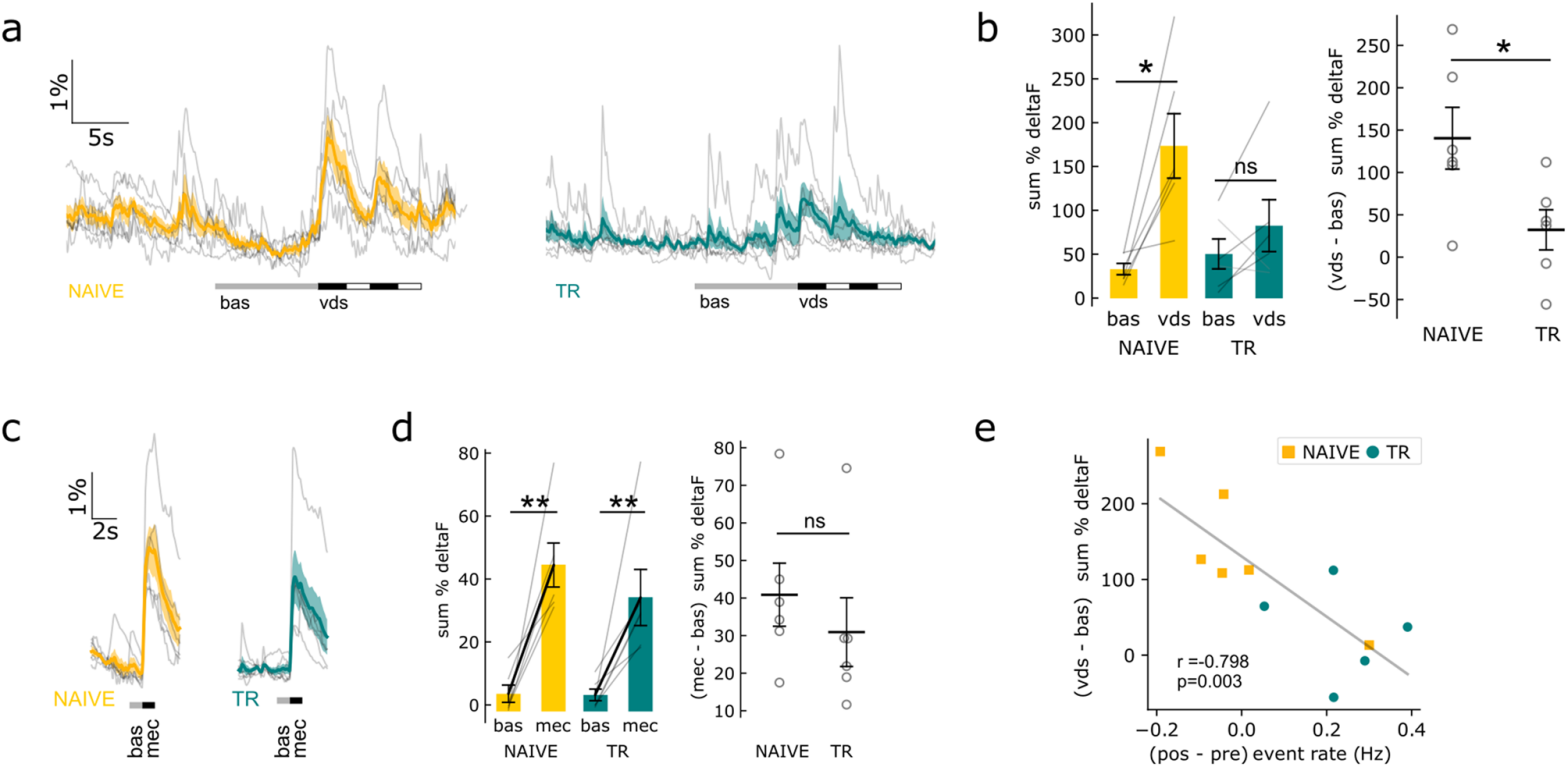
Visual danger stimulus elicited Ca^2+^ activity is reduced for trained animals. (**a**), Curves corresponding to a vds trial for NAIVE and TR animals. Thick lines and shaded areas show mean ± sem. Bars, below ΔF/F (%) curves, indicate the periods considered as basal activity and the period when the vds was active. (**b**), Summation of ΔF/F (%) during basal (bas) and vds periods (left) and the difference between activity during vds and during the bas period (right). Means ± sem are shown. (**c**) and (** d**), Idem a-b for a mechanical stimulation that consists in an “air puff” in the dorsal carapace and that serves as a control stimulus. (**f**) A negative association is display between the changes in spontaneous Ca^2+^ event rate after context presentation (Fig. 1e,f) and the activity elicited by the vds (Fig. 2b). Pearson correlation coefficient r and *p* values are shown. Post hoc paired t-tests and independent t-tests, **p < 0.01, *p < 0.05, *ns* not significant.

We next explored whether the training effect can be also observed in the long term by the unconditioned stimulus, i.e., the vds. For this, we characterized any changes in activity during a vds presentation in the same groups of animals detailed above. As expected^17,45^, vds elicited two calcium transients, one for each vds clockwise and counterclockwise excursion, clearly noticeable in the NAIVE group (Fig. 2a). In the NAIVE group, Ca^2+^ activity during the vds period was significantly higher than the spontaneous activity for the same period previously to the stimulation (i.e., basal period), while in the TR group no significant change in activity was found during the vds presentation (Fig. 2b). A two-way mixed ANOVA reported no effect for group condition (F(1,10) = 1.68, *p* = 0.224), but a period (vds vs bas, F(1,10) = 15.76, *p* = 0.002) and a group-period interaction effect (F(1,10) = 6.19, *p* = 0.032) (Fig. 2b). Post hoc t-test comparisons revealed a significantly higher activity during vds compared with basal activity for the NAIVE group (one-tailed t-test, t(5) = 3.85, *p* = 0.012), but not for the TR group (t(5) = 1.36, *p* = 0.116).

We further investigated the Ca^2+^ activity elicited by a different stimulus, a mechanical stimulation that consisted of an “air puff” at the dorsal carapace. Both groups displayed noticeable Ca^2+^ transients elicited by the mec stimulus (Fig. 2c). A two-way mixed ANOVA disclose no effect for group condition (F(1,10) = 0.916, *p* = 0.361), a period effect (mec vs. bas, F(1,10) = 33.476, *p* = 0.0002) and no group-period interaction (F(1,10) = 0.642, *p* = 0.441) (Fig. 2d). Thus, both NAIVE and TR groups elicited significant activity by mec stimuli (one-tailed t-test, NAIVE: t(5) = 4.87, *p* = 0.0046; TR: t(5) = 3.38, *p* = 0.0098) (Fig. 2d).

These results suggest that long-term training reduced the calcium transients elicited by vds in TR crabs. Furthermore, we tested whether changes in event rate after training context presentation (Fig. 1f) were related with the intensity of the vds elicited calcium activity (Fig. 2b). Data showed a significant negative correlation between event rate change after context presentation and vds elicited activity (Pearson r = − 0.798, *p* = 0.003) (Fig. 2e).

## Discussion

We’ve addressed the role of *N. granulata* MB-ls in an aversive associative long-term memory reactivation and found that network’s MB-ls can be modulated by the presentation of a reminder. Specifically, we found in trained crabs that Ca^2+^ events rate was increased after the presentation of training-context one day after training. The presentation of trained aversive stimulus embedded in the training context triggered, paralleling the paradigm’s behavioral output, reduced Ca^2+^ MB-ls transients in trained animals *vs.* naïve ones.

In terms of the identity of MB-ls and present functional data, after our group’s initial description that Neohelice’s MB-ls play roles in associative short-term memory processes^17^, other studies have proposed that such structures may be the equivalent to a neuropil described in a Stomatopod, the “reniform” body^28^. The center initially proposed by us as the structure that might resemble the reniform body in *N. granulata*^18^, was then described as a region of a crab’s mushroom body homologue that presents unique transformations from the ancestral ground pattern^27^. In other words, the MB-ls and the “reniform” body centers described in *N. granulata*^17,18^ were described in other true crabs using swapped identities^27,29,74^. Certainly, the characterization of the complex net of neuropils in the lateral protocerebrum, where the mushroom bodies are situated, of Brachyura continues to be a challenge^75^. Our recent new neuroanatomical and immunohistochemical data^18^ show that the MB-ls from *N. granulata* (and possibly all true crabs) exhibit an ensemble of several traits that comprise many characteristics proposed for centers of integration like mushroom bodies^4,13,76^. Added to the previous functional evidence^17^ they show that the MB-ls (a) is multimodal, (b) the output regions exhibit stimulus-specific activity^18^, and that (c) neuronal activity reflect context-associative components of memory processes in the short term^17^, we considered that the debate regarding the identity of these true crab's centers is open (discussed in^18,29^). The present study adds new functional evidence of the expected MBs' cognitive functions. Here, results suggest MB-ls plastic changes reflect long-term reactivation of an associative memory. Both a change in its spontaneous activity after training context-presentation as well as by the response to the trained visual danger stimulus were observed (Figs. 1 and 2). Furthermore, the expected integrative complexity of insects’ mushroom bodies structures^1,3^ has receiving a clear support by our data description of characteristic Ca^2^^+^ activity spatial patterns in the MB-ls of *N. granulata* (Supplementary Information 1 Fig. S4). Previously, we have observed overlapping but distinct spatial patterns for activity elicited by the vds or the mec stimulation in the “trauben-like region”, a proposed output region of the MB-ls^18^. After inspection of the present data in the calyx-like region, the proposed input region^18^, we also found different spatial patterns. K-mean clustering of the Ca^2+^ spatial activity patterns for each event revealed recurrent spatial configurations (Supplementary Information 1 Fig. S4, see Material and Methods). The spatial activity patterns triggered by vds and mec stimulations correspond to one of the different patterns of the registered animal during the spontaneous activity (9 of 11 crabs) (Supplementary Information 1 Fig. S4d). The recurrence of these configurations suggests that Ca^2+^ activity would be underlined by a network of different neuronal populations and/or neuronal processes, modulated by segregated multimodal inputs, as previously proposed for MBs calyces^1,3,77^. These findings open a way to speculate that spatial activity patterns triggered by vds reverberate in subsequent spontaneous activity patterns after training and/or memory reactivation sessions, as it was hypothesized^78,79^. Future experiments are still needed to further test this hypothesis.

In the framework of the long-term associative nature of this aversive memory trace, we previously proposed that the Neohelice’s MB-ls, like insects^1^, should show long-term neuronal changes underlying the context-stimulus association of the acquired information^17^. In Neohelice, the aversive memory under study involves an association between the training-context and the VDS^21,36,38^. During testing, memory expression is behavioral revealed by a decrease in activity upon the VDS presentation. This reduction results from an increased number of animals displaying freezing instead of escaping responses when the VDS was presented within the training-context^40^. Extensive evidence has shown that the *lobula* giant neurons play a role in short- and long-term memory after VDS stimulation. These neurons spread through integrative visual neuropil called *lobula*, and project to other parts of the protocerebrum, including the MB-ls^17,21,80,81^. *Lobula* giant neurons support the “what” but not the “where” of this memory; their reduced responses to the VDS in the long-term are context-unspecific. The mushroom bodies have been proposed to link learned contents within a contextual framework^1^, and the MB-ls was already proposed as the expected structure for context association^53^. The training protocol in which the change in the illumination of the training-context precedes the VDS by a few seconds produces a non-typical Pavlovian conditioning since changes in the conditioned response are mainly displayed in response to the VDS (the US)^44,82^ (Material and Methods, *Stimuli and contexts* Section). Strong-trained animals (15 trials) with this called contextual conditioning render context-specific memory expression in the short and the long-term^17,36,82^, corroborating that the reduced animals’ response to VDS in trained crabs is complicated to be explained by non-associative memory processes. Recently, MB-ls calcium signal reduction was described to be triggered by VDS both during training and short-term test sessions. Like the behavioral performances evaluated in this memory paradigm that are context-dependent^21,36,38,40,44^, MB-ls Ca2 + signal response to the VDS was recovered when the context was modified between training and testing, revealing that the plastic changes in the MB-ls structure reflect the context-specific feature of this visual-aversive associative memory already in the short term^17^.

To the best of our knowledge, results presented here are first evidence regarding crustacean’s brain centers that might be involved in associative long-term memory traces: results support that memory reactivation in long-term (24 h) by the training context presentation induced a change in the spontaneous activity of trained, but not naïve animals (Fig. 1e,f). It is widely accepted that long-term memory consolidation -among many of the general principles of memory organization conserved throughout evolution in bilateral animals^40,83–85^—depends upon de novo protein synthesis^86^. In the *N. granulata* associative paradigm, memory consolidation, reconsolidation and extinction involve de novo protein synthesis^21,40,41^. During consolidation, cycloheximide was shown to interfered with the context-signal associative component of long-term memory generated by spaced training^69^. Between the several elements that show that this memory processes are hard to be interpreted as non-associative process is that the training-context presentation without the VDS (the same reminder structure used here) is a necessary condition to trigger the reconsolidation process^22,50^. For instance, the disruption of the reconsolidation process by amnesic agents, like cycloheximide, results in responses to the VDS that are indistinguishable between trained and control animals at the subsequent testing sessions^50^. To support the spontaneous activity increase in the MB-ls of trained animals is part of a process that went through associative memory consolidation, we added here the evaluation of whether the protein synthesis inhibitor cycloheximide can block this long-term change when administered immediately after training, as it was observed at the behavioral level in previous studies^69^. Specifically, cycloheximide-treated animals underwent the same experimental conditions previously used for trained animals. Results suggested that de novo protein synthesis was required for the expression of long-term changes of MB-ls spontaneous activity triggered by the training-context presentation (Supplementary Information 1 Figs. S5 and 2 S3). Furthermore, cycloheximide experiments also supported the existence of an association between the context and the visual danger stimuli (Supplementary Information 1 Fig. S5): consistently with previous reports^17^, presentation of the context plus the VDS elicited an enhancement of Ca^2+^ MB-ls transient for both naïve and cycloheximide treated, but not in trained crabs (Fig. 2a,b and Supplementary Information Fig. S5). Two other subjects support the notion of the associative mnesic nature of the changes in MB-ls’ Ca^2+^ activity described in this study. In Neohelice post-training cycloheximide is known to interfere with the associative but not with non-associative components of the aversive long-term memory^69^. Additionally, negative correlation between responses to the VDS with the activity elicited only by a training-context presentation (Fig. 2e and Supplementary Information 1 Fig. S5), suggested another facet of the associative mnesic nature of the changes in MB-ls’ Ca^2+^ activity described in this work. Future studies to compare untrained (i.e., context presentation only) versus trained animals are still needed to add additional support to the hypothesis that the different MB-ls’ calcium responses in trained and naïve crabs could be interpreted as non-associative process.

During the presentation of the training context, we could not find significant changes in the spontaneous activity in both the naïve and training groups (Supplemental Information 1 Fig. S3). Additionally, we could not observe that, in the proposed calyx of the MB-ls, the presentation of training context itself initiates Ca^2+^-transients (Supplemental Information 2 Fig. S1–3, ctx segments) like the induced by the appearance of visual or mechanical stimuli. Likewise, we could not describe consistent Ca^2+^-transients for the training-context presentation also in the proposed output region of the MB-ls^17^. The differential MB-ls reactions between context presentations versus visual and mechanical stimuli highlights that the Ca^2+^ neuronal signals described in these studies have various degrees of specificity.

Memory retrieval, or the expression of reactivated memories, is modulated by internal states^56,87,88^. The encoding of these internal states can include the modulation of spontaneous activity of the nervous system^30^. Spontaneous oscillations of the membrane potential or intracellular calcium concentrations of a wide range of frequencies (0.1–40 Hz in invertebrates, 1–200 Hz in vertebrates) have been described^35,89–92^. These oscillations modify behaviors and have been proposed to take part in working memories, and in the processes of learning, consolidation and retrieval^93,94^. Indeed, tyrosine hydroxylase immunoreactivity is present in Neohelice’s MB-ls^18^. There is evidence relating monoamines control with oscillatory activities that reflect specific internal states^30^. Dopamine levels present slow spontaneous oscillations in specific areas of the rodent brain, including the hippocampus^30,95^, and have been shown to intervene in the processing of new contexts, rewards, motivation, and learning and memory^95^. In Drosophila MBs, the spontaneous activity of dopaminergic neurons might reflect the internal state of the animals, modulating cognitive processes such as decision-making and memory consolidation^30,35^. After training, dopaminergic neurons that innervate MBs initiate low-frequency oscillations of Ca^2+^ activity (~ 0.1 Hz) which were associated with long-term memory formation; altering flies’ internal state by severe starving results in the lack of initiation of these slow oscillations and also blunting of long-term memory^35^. Results showed here support that long-term memory reactivation, by the presentation of the training context, induces a change in the spontaneous Ca^2+^-dependent activity of trained crabs in the MB-ls (Fig. 1e,f). This change in spontaneous activity might reveal the appearance of a specific internal state in *N. granulata* that modulates the concurrent cognitive processes.

Besides the support for the putative roles of the MB-ls in associative memories, these results highlighted plausible experimental approaches to examine mechanisms underlying the reevaluation of past experiences based on current information. First, the context-reminder used here (Fig. 1b) contains the parametric structure to trigger memory reconsolidation^22,36^; known to depend on prediction error between real and expected experiences during the reminder sessions^22,56,60,61^. Although the neural mechanisms that allow prediction error to update memories remain unknown, there has been increasing evidence of the role of hippocampus activation and its neuromodulation by dopamine and acetylcholine^96^. It is possible that the surged Ca^2+^ activity we described here after a context-training presentation would signal the labilization of the consolidated memory trace that enters reconsolidation. The study suggested above, comparing changes in MB-ls Ca^2+^-activity in training groups, where memory is first reactivated by the reminder that initiates reconsolidation (training-context presentation) *vs*. the reminder that does not start the process (training-context presentation + VDS), are needed to further understand the mechanism of labilization of this consolidated memory^22,56^. Second, the changes in spontaneous activity might reveal the appearance of specific (emotional^97^) internal state^30^. Our current working hypothesis, in both behavioral and calcium imaging experiments designs, posits that the unfolding of these internal states at testing sessions are determinants of the behavioral expression of reactivated memories. It’s our lab’s view that, during reconsolidation, endogenous neuromodulators triggered by concurrent experiences might control the probability that reactivated memory to guide behavior. Various studies show that memory reactivation—labilization occurs even in behavioral unexpressed memories^55,56,58,98^. Based on the Wagner´s Affective Extension of Sometimes Opponent Processes (AESOP) model—where any US is represented by separate, independent, sensory and emotive components^99–101^—our hypothesis proposes that these changes in internal states are the unfolding of the emotive components. According to AESOP, this unfolding of the emotive component is a critical modulator of the behavioral responses occasioned by the reactivation of the sensory component of the memory trace^100^. The results here presented open a way to explore the neuronal mechanisms through which the changes in the internal states, triggered by the reactivation of the trace, will be crucial in determining the behavioral expression of the reactivated associative memories^56,102^.

## Supplementary Information

Supplementary Information 1.

Supplementary Information 2.

Supplementary Information 3.

## Data Availability

All data generated or analysed during this study are included in this published article and its supplementary information files.
