# Supplementary material for: Contextual memory reactivation modulates Ca2+-activity network state in a mushroom body-like center of the crab N. granulata
Source: Sci Rep. 2022 Jul 6;12:11408. doi: 10.1038/s41598-022-15502-1 (PMC9259570; doi:10.1038/s41598-022-15502-1)
Supplement: Supplementary file 2 — Supplementary Information 2. [file 41598_2022_15502_MOESM2_ESM.pdf]

Supplementary Information 1

Contextual memory reactivation modulates Ca^{2+}-activity network state in a mushroom body-like center of the crab *Neohelice granulata*

Francisco Javier Maza, Francisco José Urbano, Alejandro Delorenzi

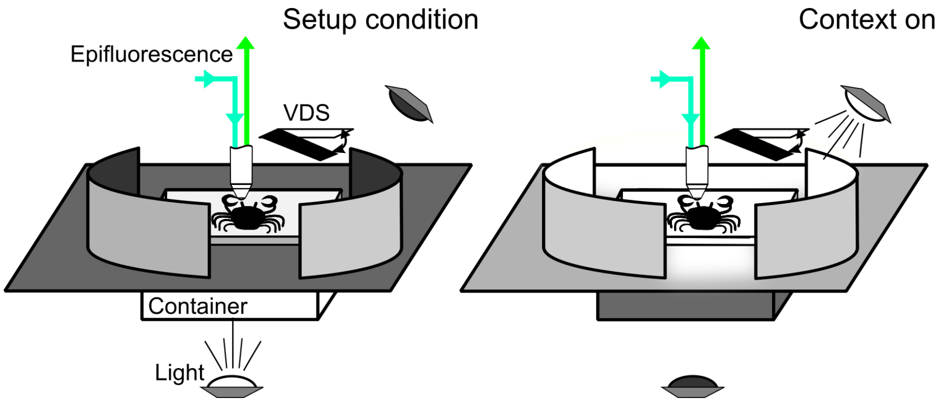

Supplementary Information 1. Figure S 1. Recording setup. Cartoon showing the relative location of the crab under the recording setup. Phasic presentation of the training context is done by a change in setup illumination. Crab is held partially submerged in a container with brackish water. VDS: visual danger stimulus.

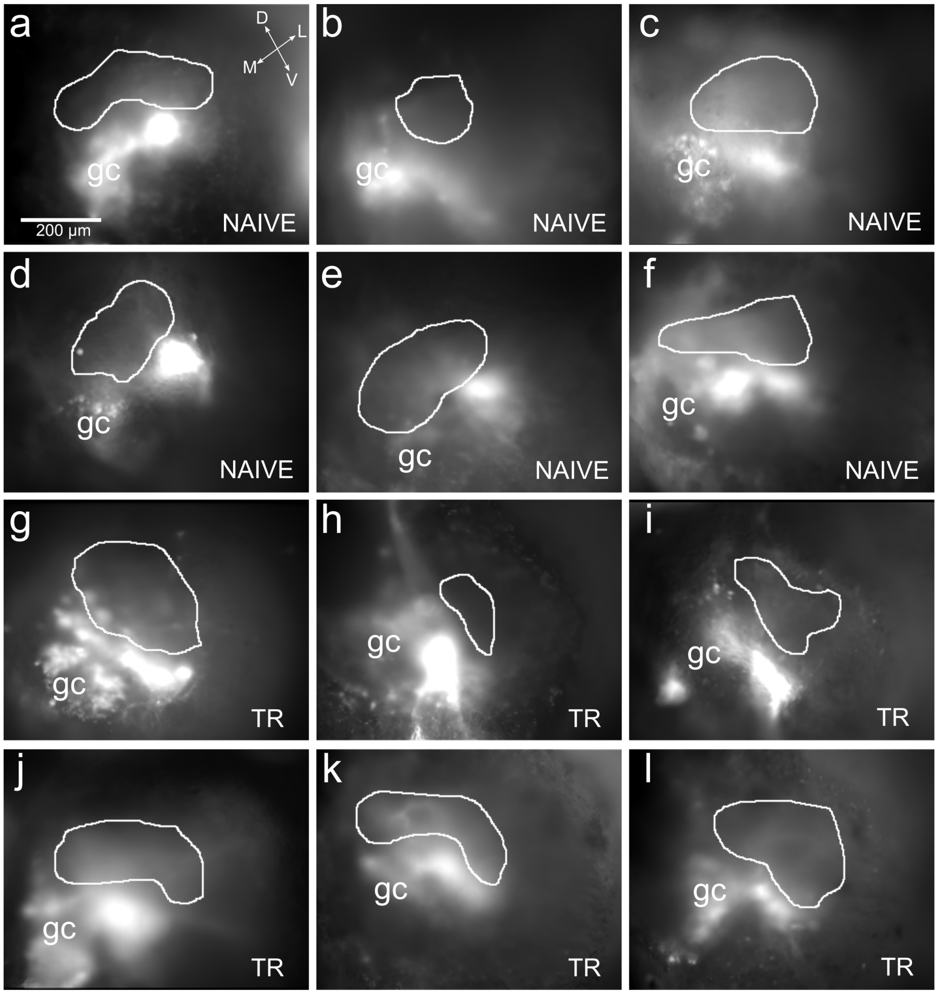

Supplementary Information 1. Figure S 2. Representative calcium imaging frames. Fluorescence pictures depicting the region of interests (ROIs, encircled). ROIs were selected manually and comprises areas of the MB-ls that are close to its intrinsic globuli cells somata (gc). High fluorescence regions correspond to the site where the calcium sensitive dye (Calcium Green-1 dextran) was stabbed. **a-f**, untrained group (NAIVE). **g-l**, trained (TR) group. Abbreviations: D: dorsal, L: lateral, V: ventral, M: medial.

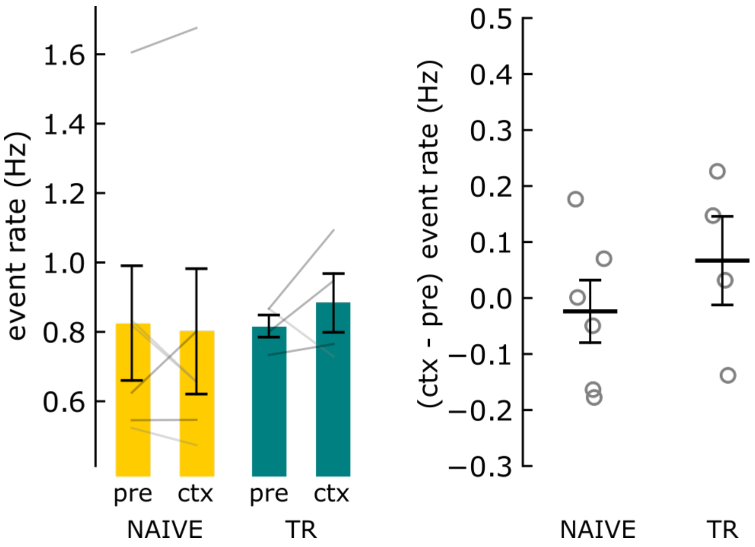

Supplementary Information 1. Figure S 3. Spontaneous activity during context presentation was not significantly different from pre context. Event rate (Hz) for NAIVE and TR animals during pre and ctx periods (left) and difference between ctx and pre period (right). Grey lines and circles correspond to individual animals. Means ± sem are shown.

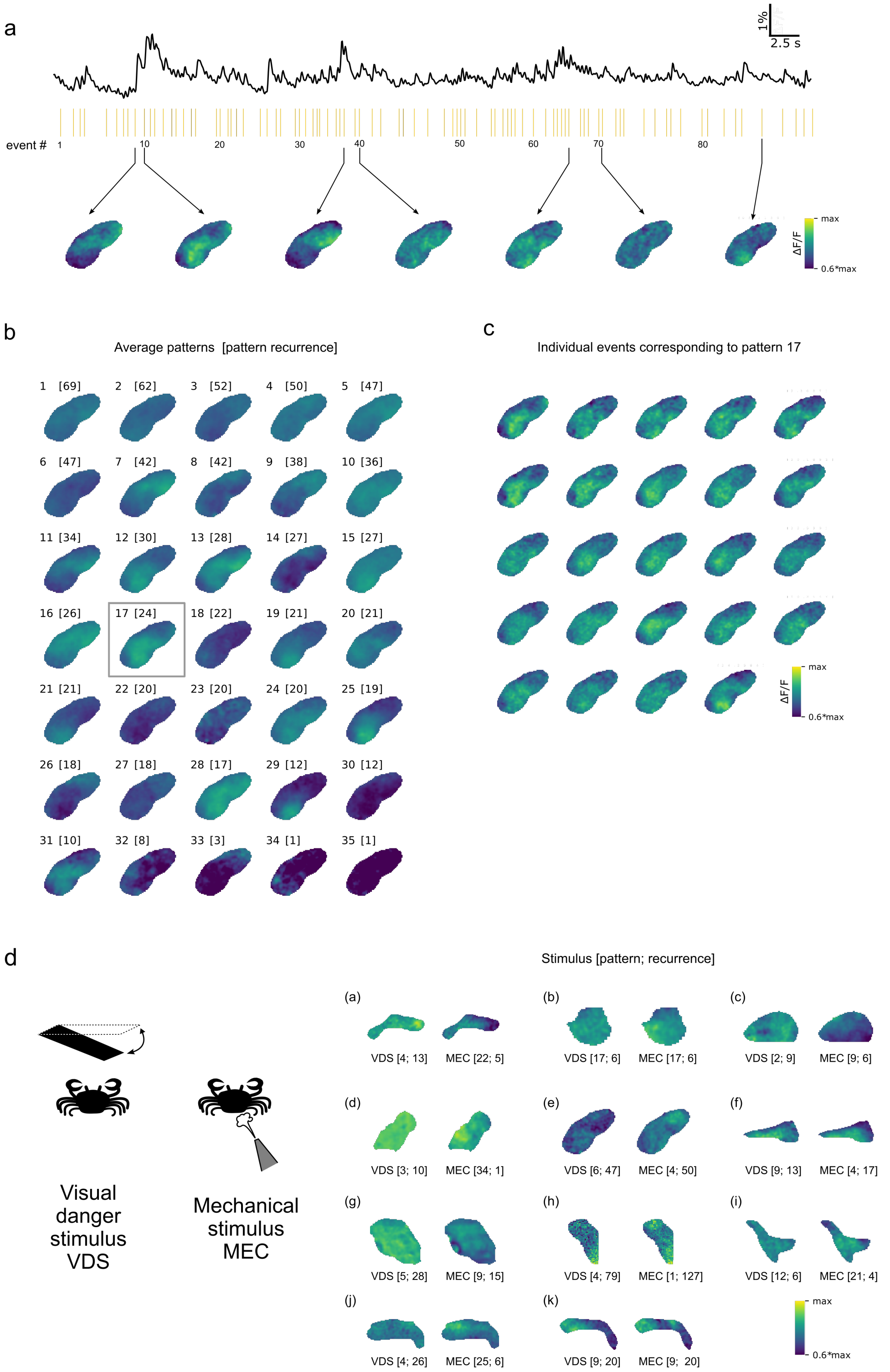

Supplementary Information 1. Figure S 4. Fluorescence activity patterns distribution. a, Examples of Ca2+-mediated spatial patterns of selected events for an animal. Mean spatial activity ΔF/F (%) during peak (±1 frame) is shown in color-coded images below the events. Spatial patterns images corresponding to the average of the event peak frame ±1, were normalized to the maximum pixel value inside the ROI. Patterns considered pixel relative intensities. Patterns were obtained for events in all recording segments available for each animal ("pre", "ctx", "pos", "vds", "posvds", and "mec"). **b**, All spatial patterns observed for the same animal shown in (a) after K-means clustering including spatial activity for all events. Each picture corresponds to the average of events assigned to each cluster by the K-Means algorithm. The observed recurrence, or size, of the pattern is shown in numbers within brackets. Patterns numbers were assigned from the most to the less recurrent. c, all events assigned to pattern number 17 in the same example of (a) and (b). **d**, Spatial activity during VDS or MEC stimuli for all animals and their corresponding pattern recurrence for each animal.

Protein Synthesis Inhibitor Cycloheximide After Training

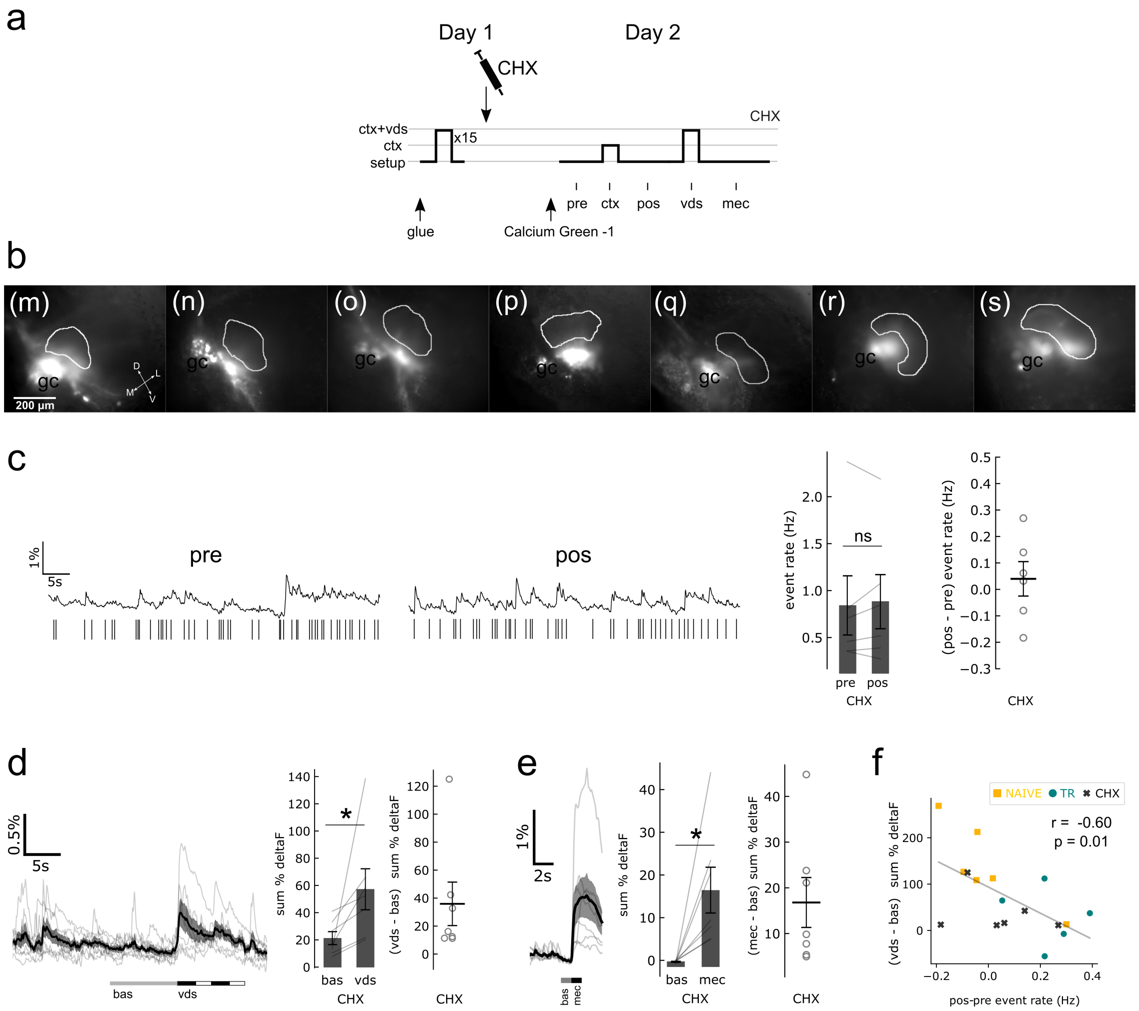

Supplementary Information 1. Figure S 5. Protein synthesis inhibitor Cycloheximide interfered with Ca^{2+} changes observed in trained animals. **a**, Experimental protocol. On day one, crabs have their left eyestalk glued in place ready to be open for recording at day two. Protein synthesis inhibitor Cycloheximide (40 μg/crab, CHX) was injected systemically immediately after training (15 trials, ISI 3 min). On day two, Calcium Green -1 dextran was applied. Recordings involved periods of no stimulation (pre, pos) and trials with context, visual or mechanical stimulation (ctx, vds and mec). **b**, Fluorescence pictures depicting the region of interests (ROIs, encircled) for each CHX animals ("m" to "s"). ROIs were selected manually and comprises areas of the MB-ls that are close to its intrinsic globuli cells somata (gc). High fluorescence regions correspond to the site where the calcium sensitive dye (Calcium Green-1 dextran) was stabbed. **c**, Left, examples of ΔF/F (%) obtained during pre and pos context presentation periods for a CHX animal. Ticks below each curve correspond to Ca2+ events. At the right, event rate (Hz) for CHX during pre and pos periods (left) and difference between pos and pre period (right). Grey lines and circles correspond to individual animals. Means ± sem are shown. No difference was found between pre and pos event rates, two-tailed paired t-test (t(5)= 0.65, p= 0.57). Events amplitudes throughout pre and pos periods were analyzed; no differences between pre and pos periods were found for events amplitudes (pos minus pre mean %ΔF/F amplitude ± sem; CHX: 0.000134± 0.0137; t-test t(5)= 0.01, p=0.99). **d**, Curves corresponding to a vds trial for CHX animals. Thick lines and shaded areas show mean ± sem. Bars, below ΔF/F (%) curves, indicate the periods considered as basal activity and vds period. At the right, summation of ΔF/F (%) during basal (bas) and vds periods and the difference between activity during vds and during the bas period. The vds elicited Ca2+ transients. One-tailed paired t-test (t(6)= 2.31, p= 0.03). **e**, Idem (d) for a mechanical stimulation that consists in an "air puff" in the dorsal carapace and that serves as a control stimulus. One-tailed paired t-test (t(6)= 3.08, p= 0.01). **f**, Correlation between the changes spontaneous Ca2+ event rate after context presentation and the activity elicited by the vds. Note a negative correlation when pooled with the NAIVE and TR groups (Figure 2e). Pooled values obtained from cycloheximide-treated animals presented lower values compared to other groups. Pearson correlation coefficient r and p values are shown. One-tailed paired t-tests, *p<0.05, ns: not significant. Means ± sem are shown. Abbreviations: gc: globuli cells, D: dorsal, L: lateral, V: ventral, M: medial.
