# Supplementary material for: Contextual memory reactivation modulates Ca2+-activity network state in a mushroom body-like center of the crab N. granulata
Source: Sci Rep. 2022 Jul 6;12:11408. doi: 10.1038/s41598-022-15502-1 (PMC9259570; doi:10.1038/s41598-022-15502-1)
Supplement: Supplementary file 3 — Supplementary Information 3. [file 41598_2022_15502_MOESM3_ESM.pdf]

## Supplementary Information 2

# Contextual memory reactivation modulates $Ca^{2+}$-activity network state in a mushroom body-like center of the crab *Neohelice granulata*

**Francisco Javier Maza, Francisco José Urbano, Alejandro Delorenzi**

# Untrained Animals (NAIVE)

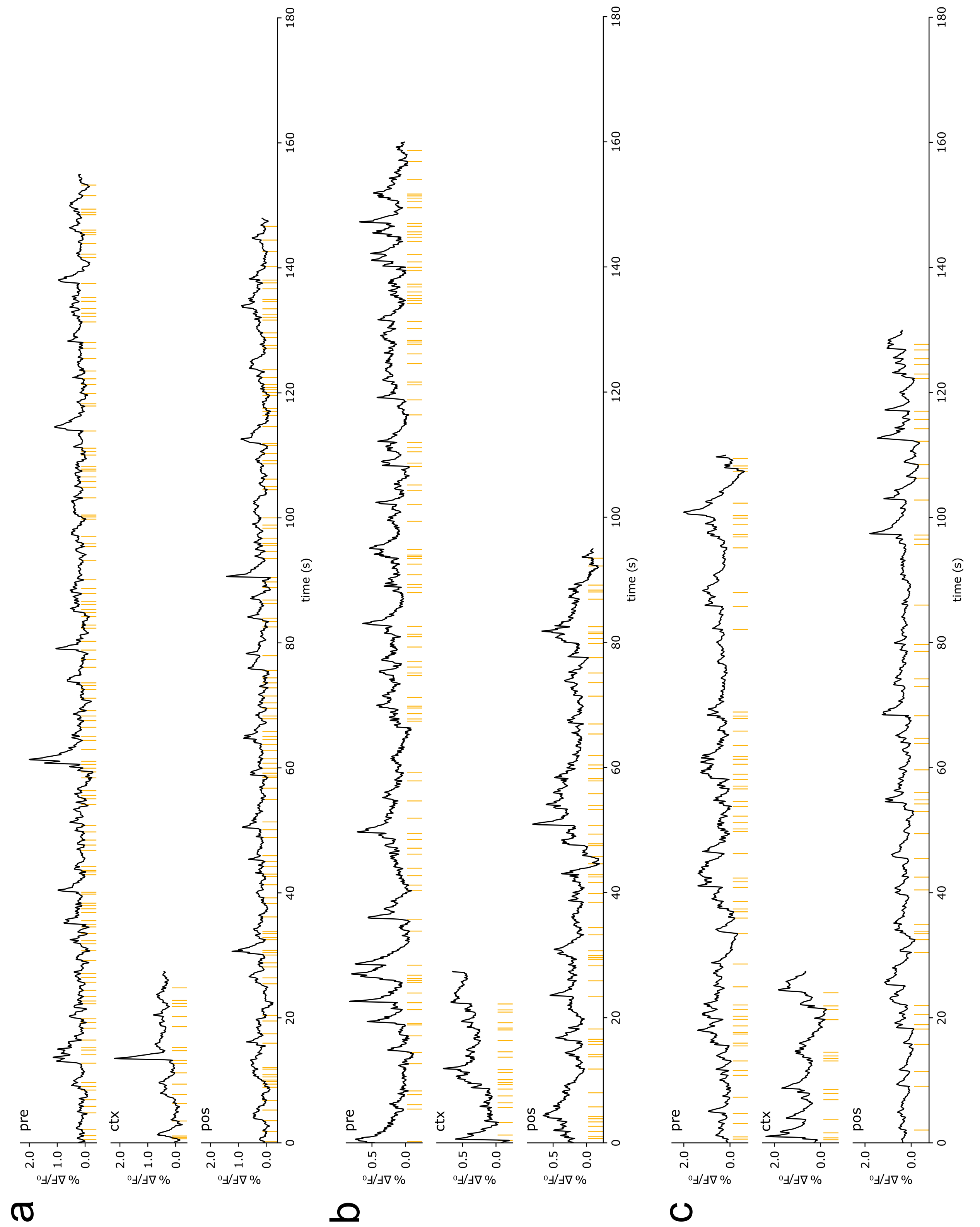


**Supplementary Information 2. Figure S 1. Spontaneous Ca2+ activity from untrained animals (NAIVE).** Calcium activity during pre, ctx and pos context presentation periods. Fluorescence dynamics as ΔF/F(%) obtained from the hemiellipsoid body while the animals rest in the setup without explicit stimulation. **a-f**, untrained group (NAIVE). Upper traces for each animal correspond to pre context presentation registers (pre). Middle traces, context presentation (ctx). Lower traces, pos context presentation registers (pos). Vertical lines indicate calcium events obtained as described in Material and Methods (Calcium events). Continues in next page.

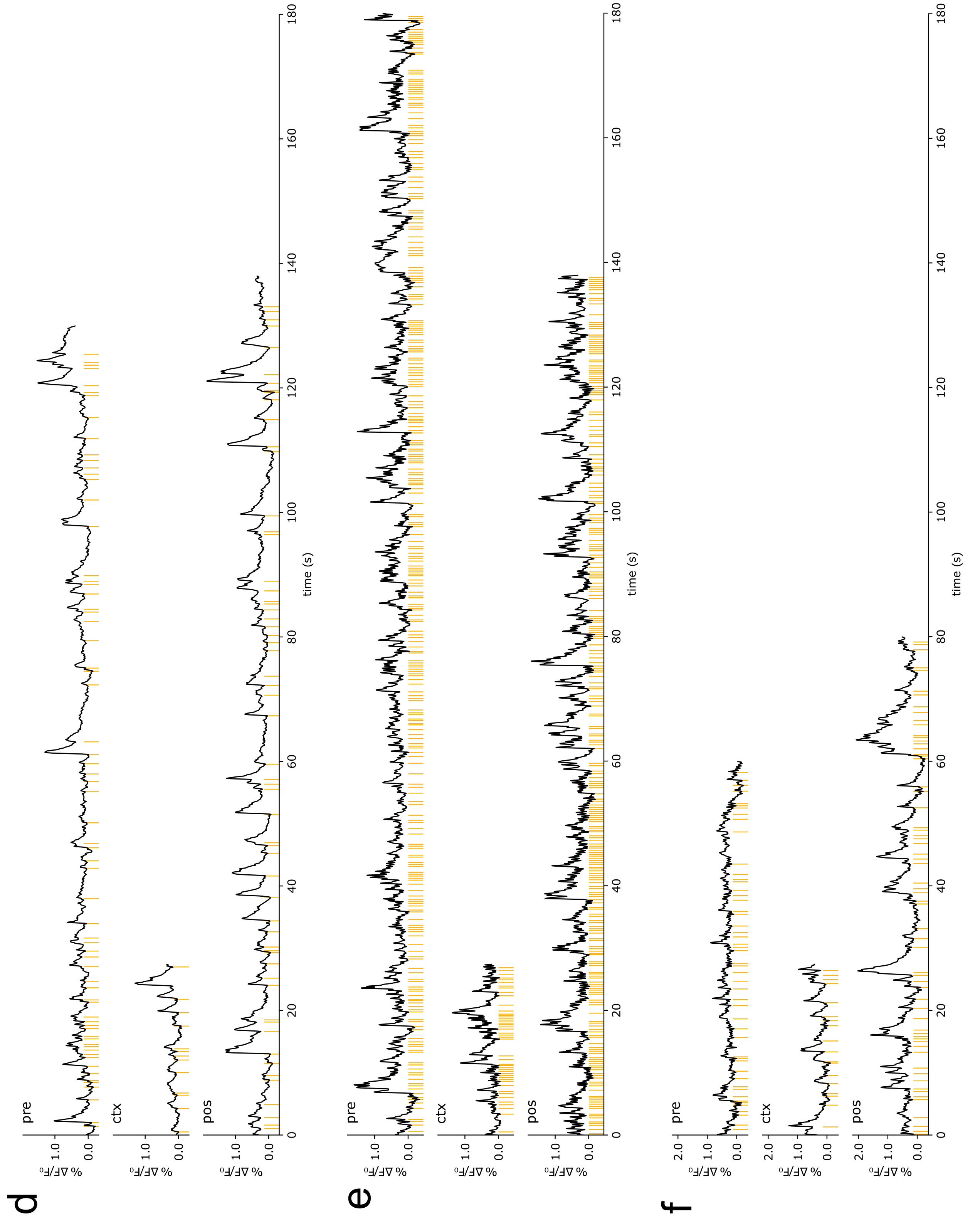


**Supplementary Information 2. Figure S 1.** (Cont.) Spontaneous calcium activity during pre, ctx and pos context presentation periods.

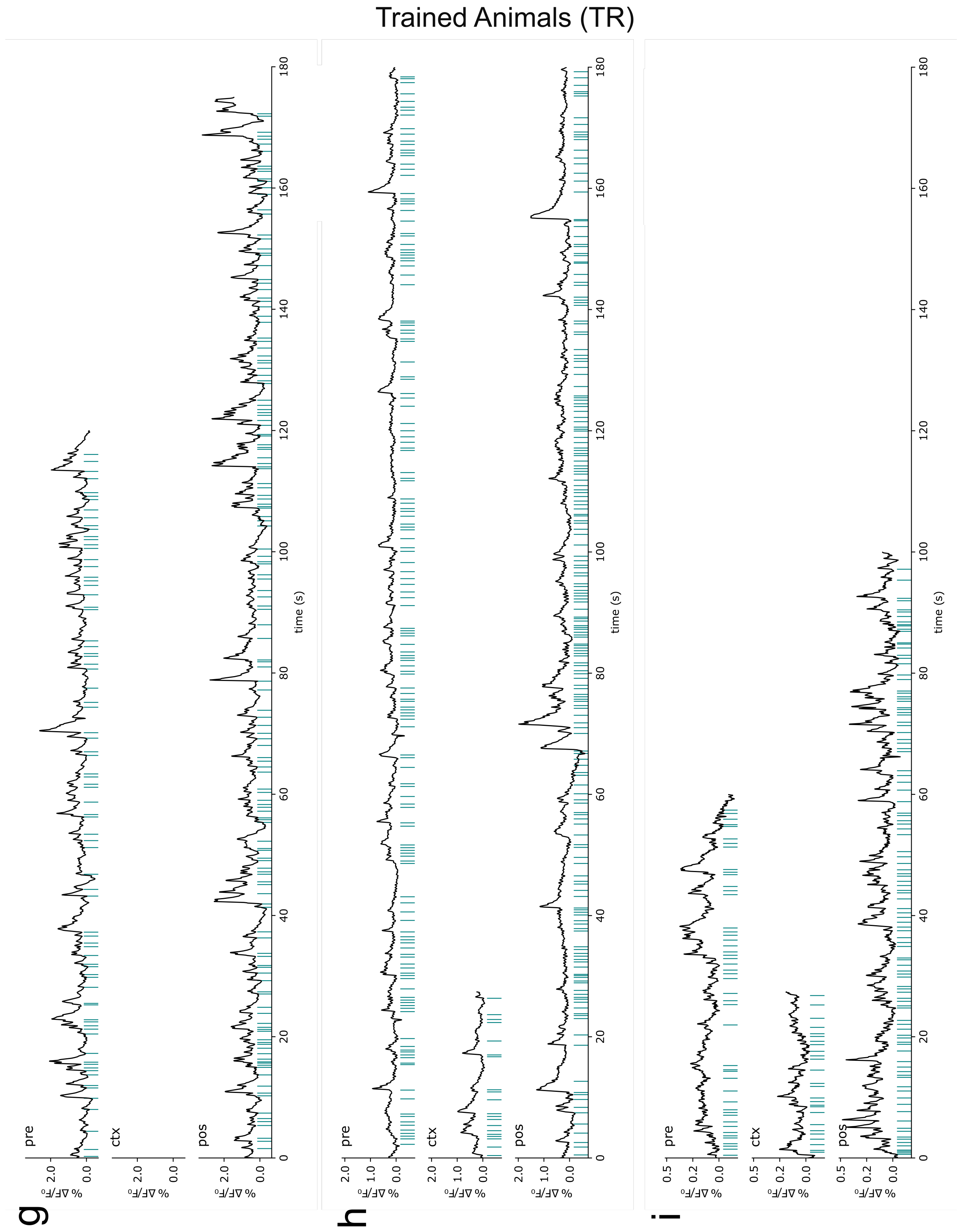


**Supplementary Information 2. Figure S 2. Spontaneous Ca2+ activity from trained animals (TR).** Spontaneous calcium activity during pre, ctx and pos context presentation periods. Idem Supplementary Figure 2.1. **g-k**, trained animals (TR). Continues in next page.

## Trained Animals (TR) (Cont.)

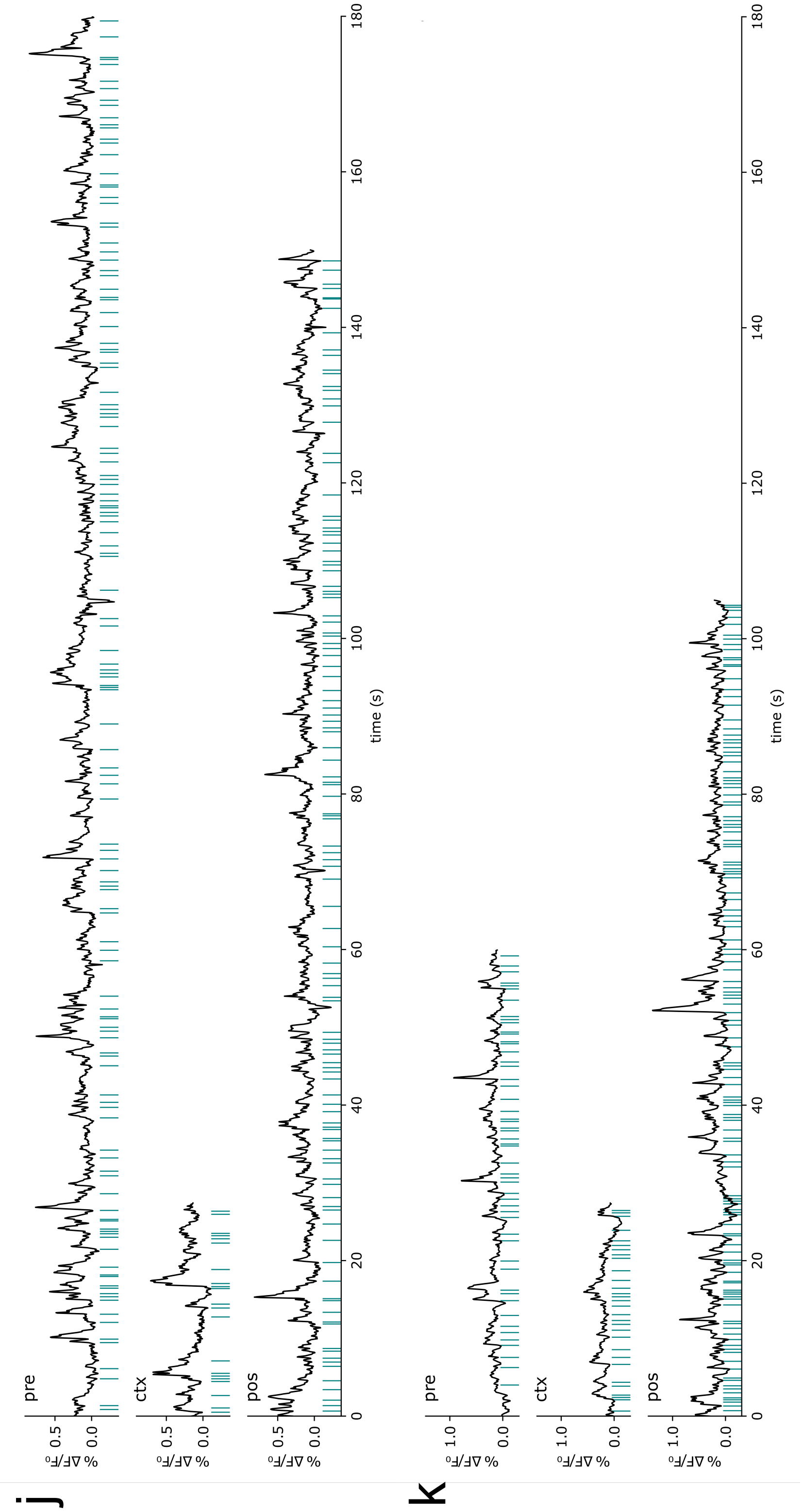


**Supplementary Information 2. Figure S 2.** (Cont.) Spontaneous calcium activity during pre, ctx and pos context presentation periods.

## Cycloheximide-Treated Trained Animals (CHX)

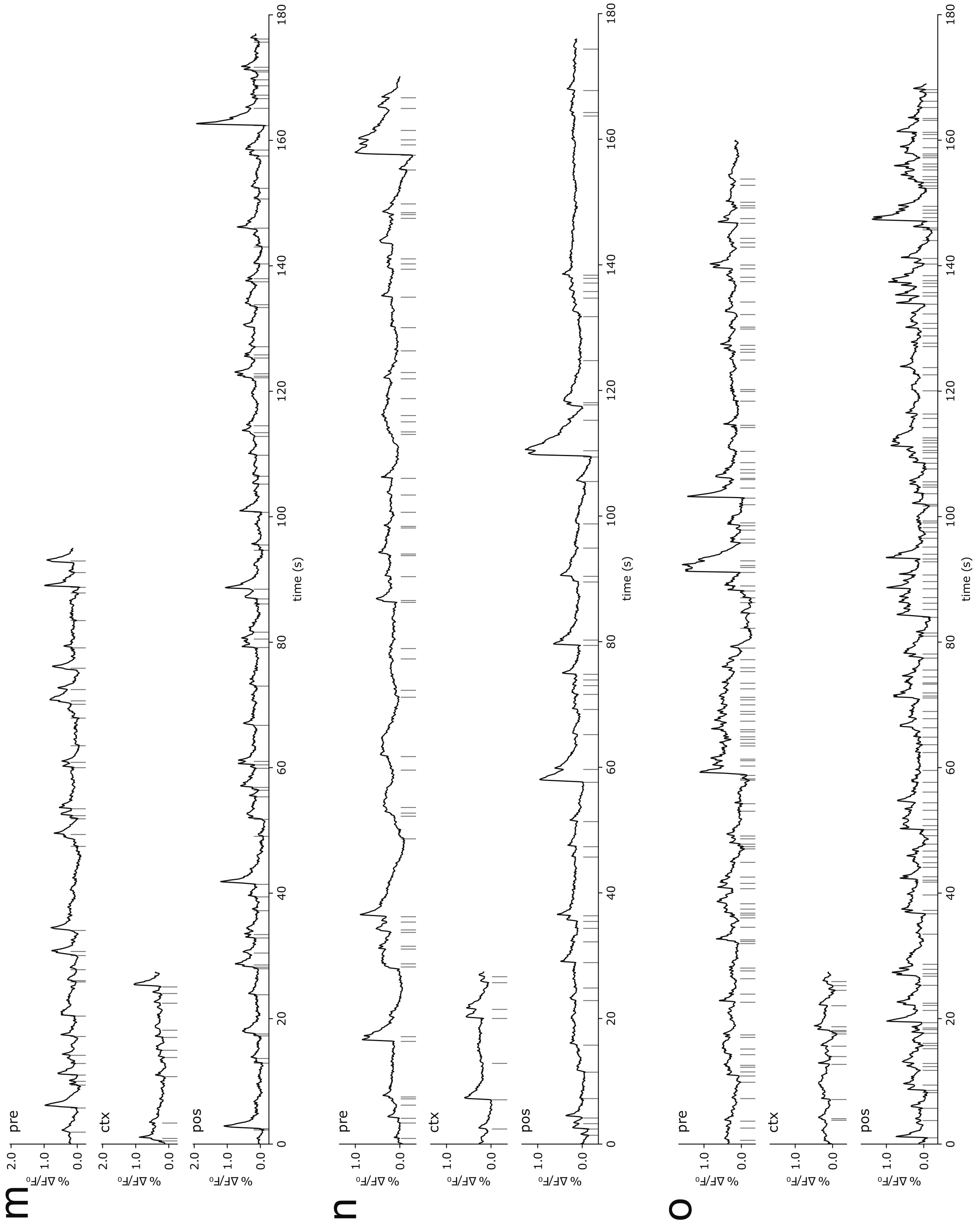


**Supplementary Information 2. Figure S 3. Spontaneous Ca2+ activity from cycloheximide-treated animals (CHX).** Spontaneous calcium activity during pre, ctx and pos context presentation periods. Idem Supplementary Figure 2.1. **m-r**, trained animals treated with cycloheximide (CHX). Continues in next page.

## Cycloheximide-Treated Trained Animals (CHX) (Cont.)

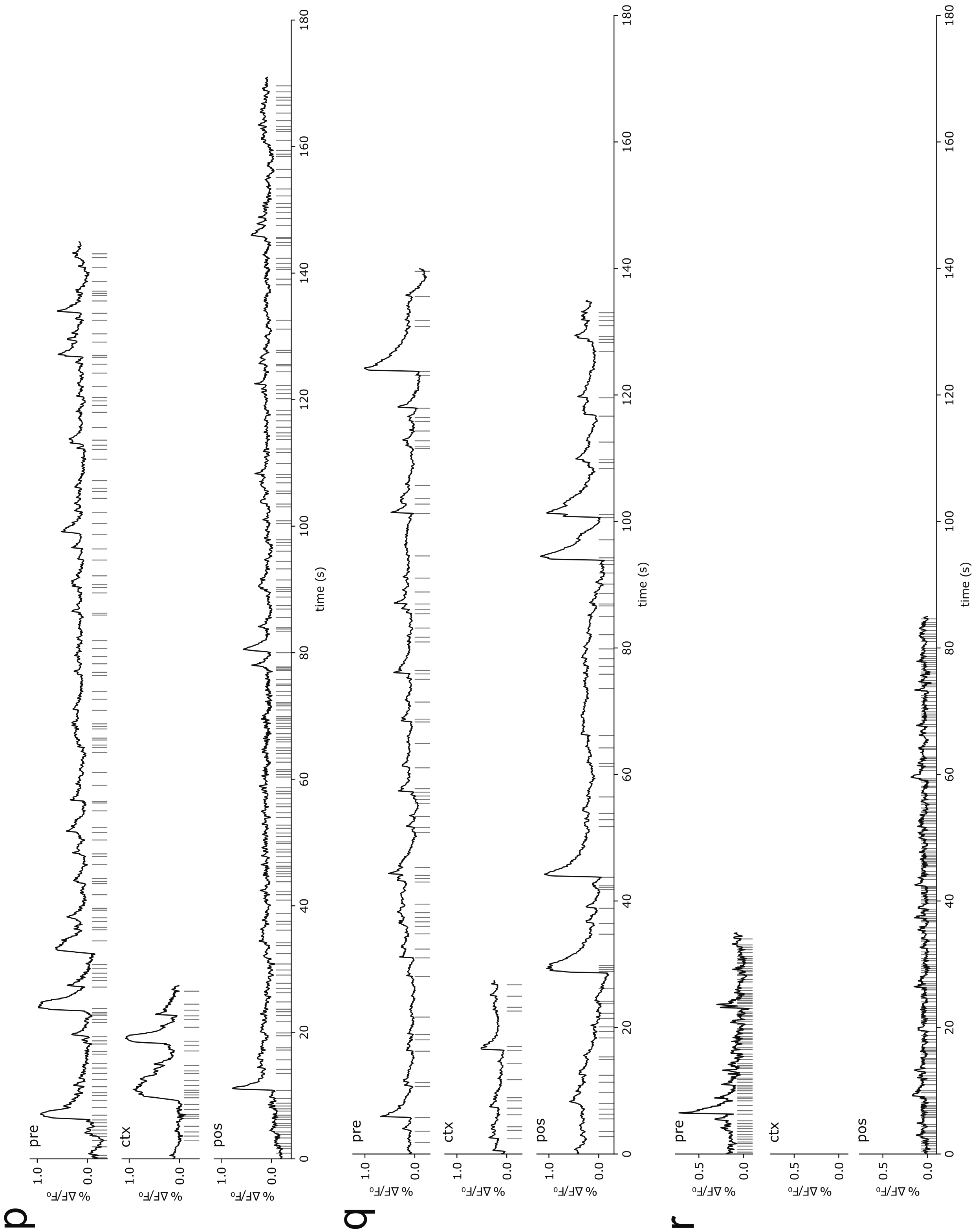


**Supplementary Information 2. Figure S 3.** (Cont.) Spontaneous calcium activity during pre, ctx and pos context presentation periods.
